# Supplementary material for: The Effects of a Bacterial Endotoxin on Behavior and Sensory-CNS-Motor Circuits in Drosophila melanogaster
Source: Insects. 2019 Apr 22;10(4):115. doi: 10.3390/insects10040115 (PMC6523965; doi:10.3390/insects10040115)
Supplement: Supplementary file 1 [file insects-10-00115-s001.zip › insects-479312-Supplementary/behaviors defined-INSECT 2019.pptx]

## Slide 1
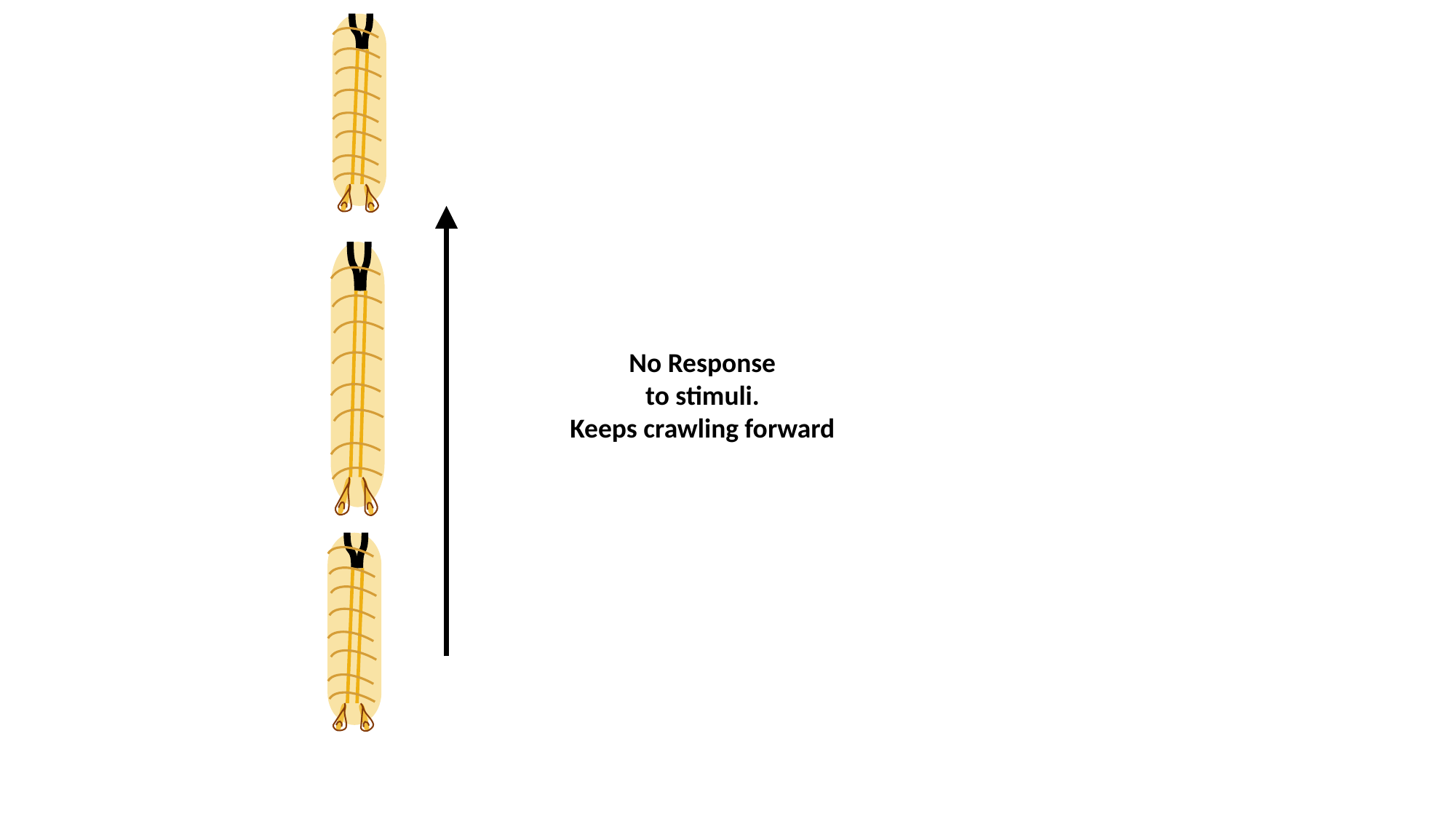

No Response
to stimuli.
Keeps crawling forward

## Slide 2
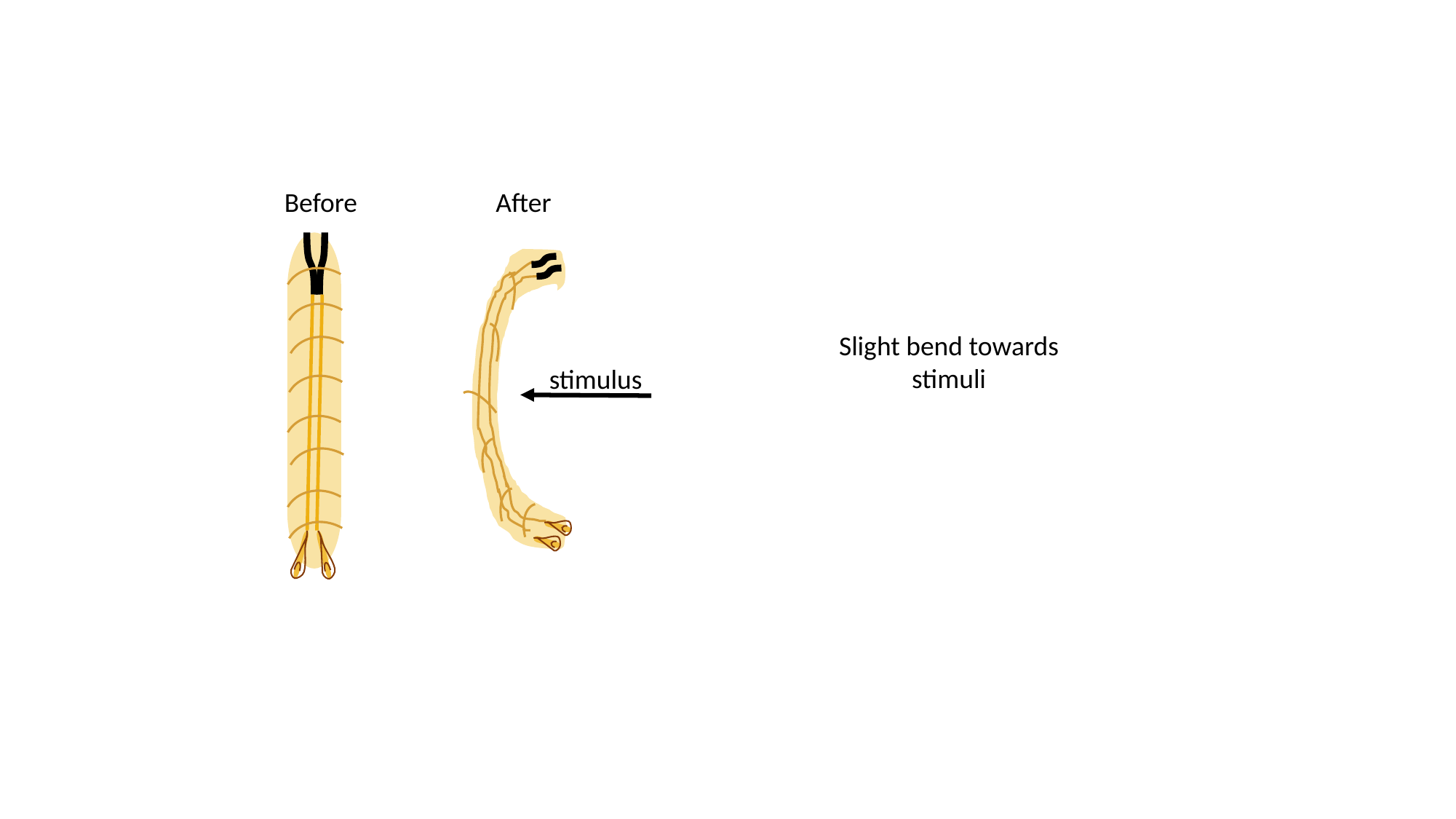

After
Before
Slight bend towards
stimuli
stimulus

## Slide 3
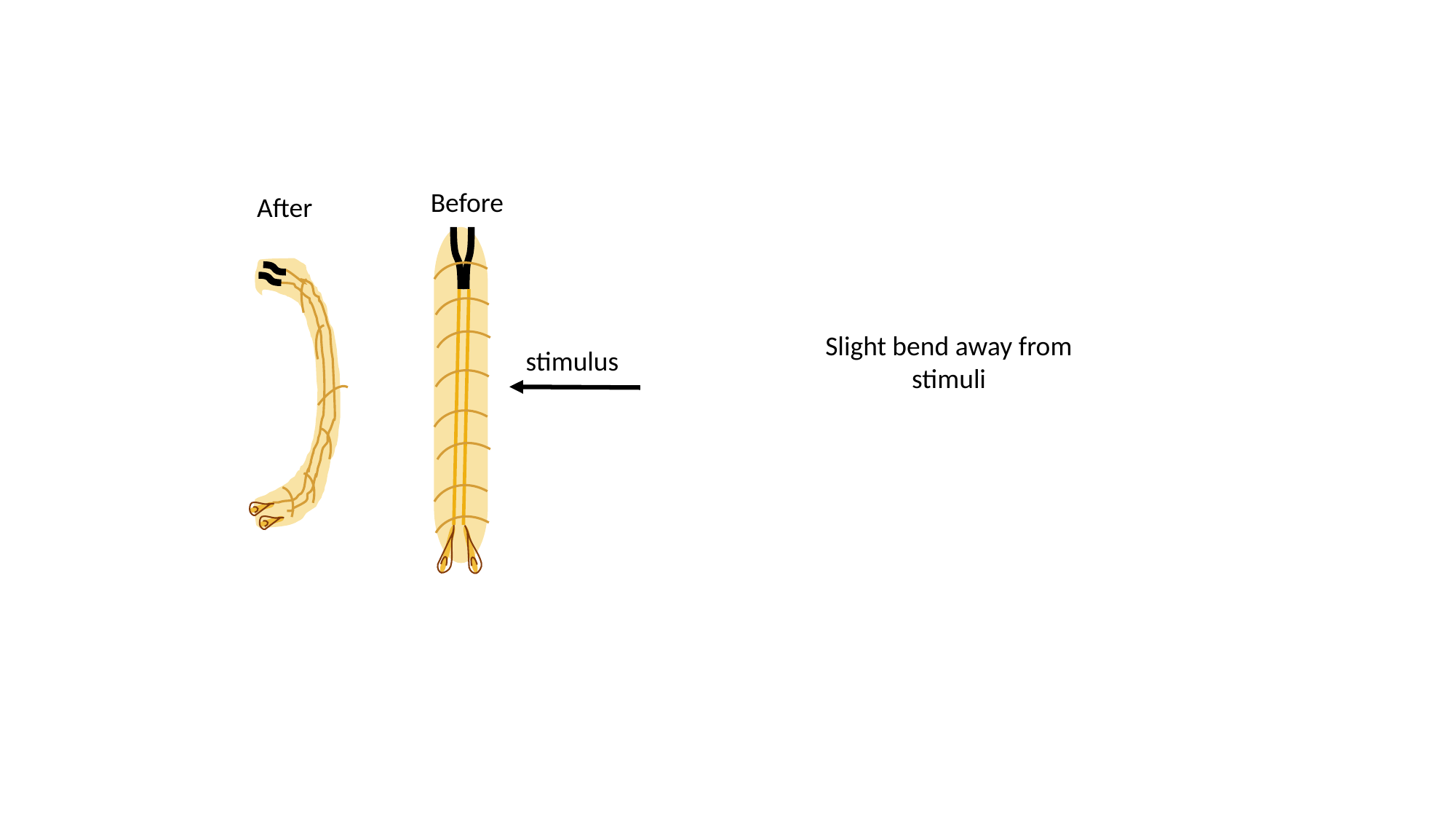

Before
After
Slight bend away from
stimuli
stimulus

## Slide 4
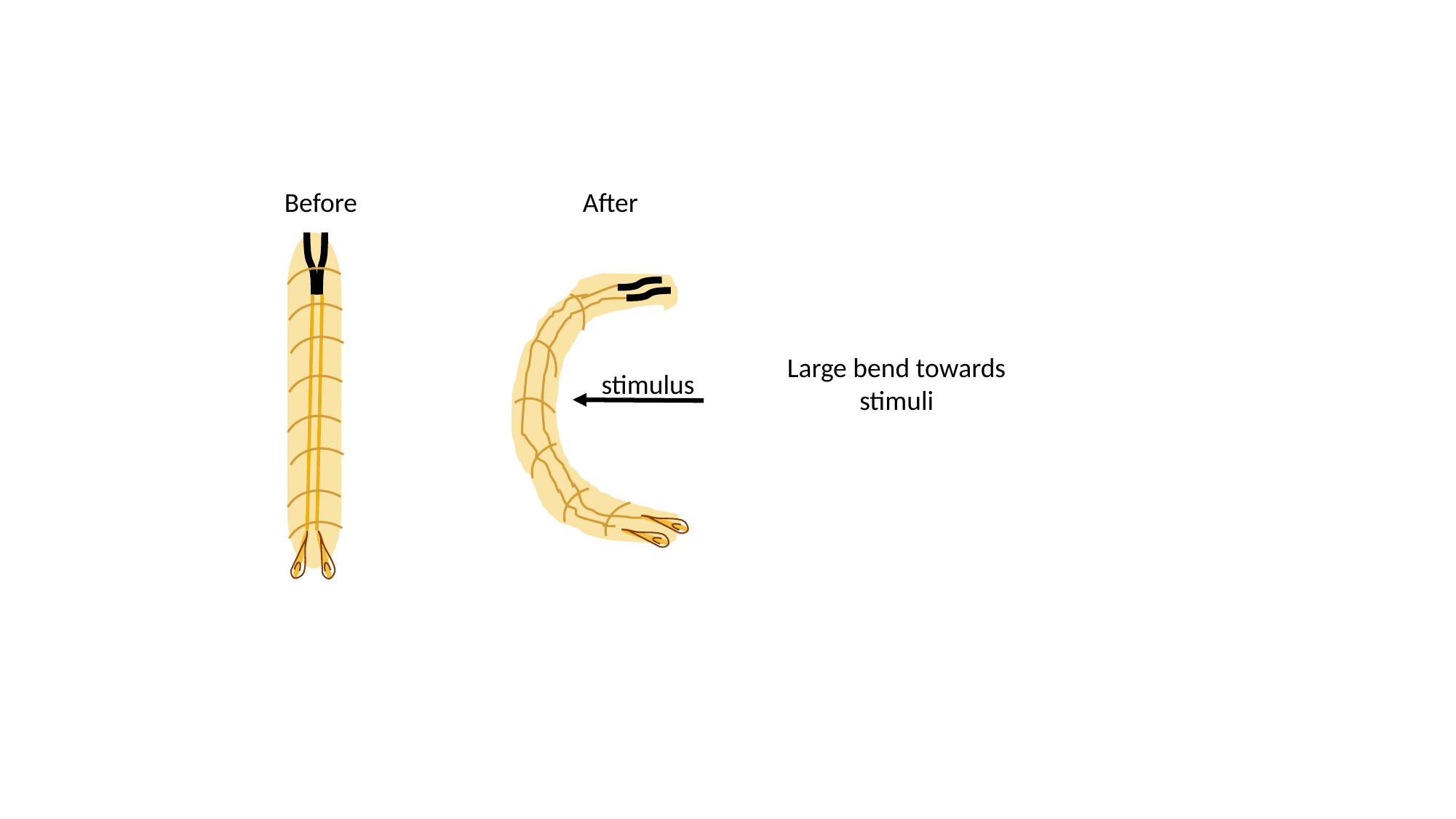

After
Before
Large bend towards
stimuli
stimulus

## Slide 5
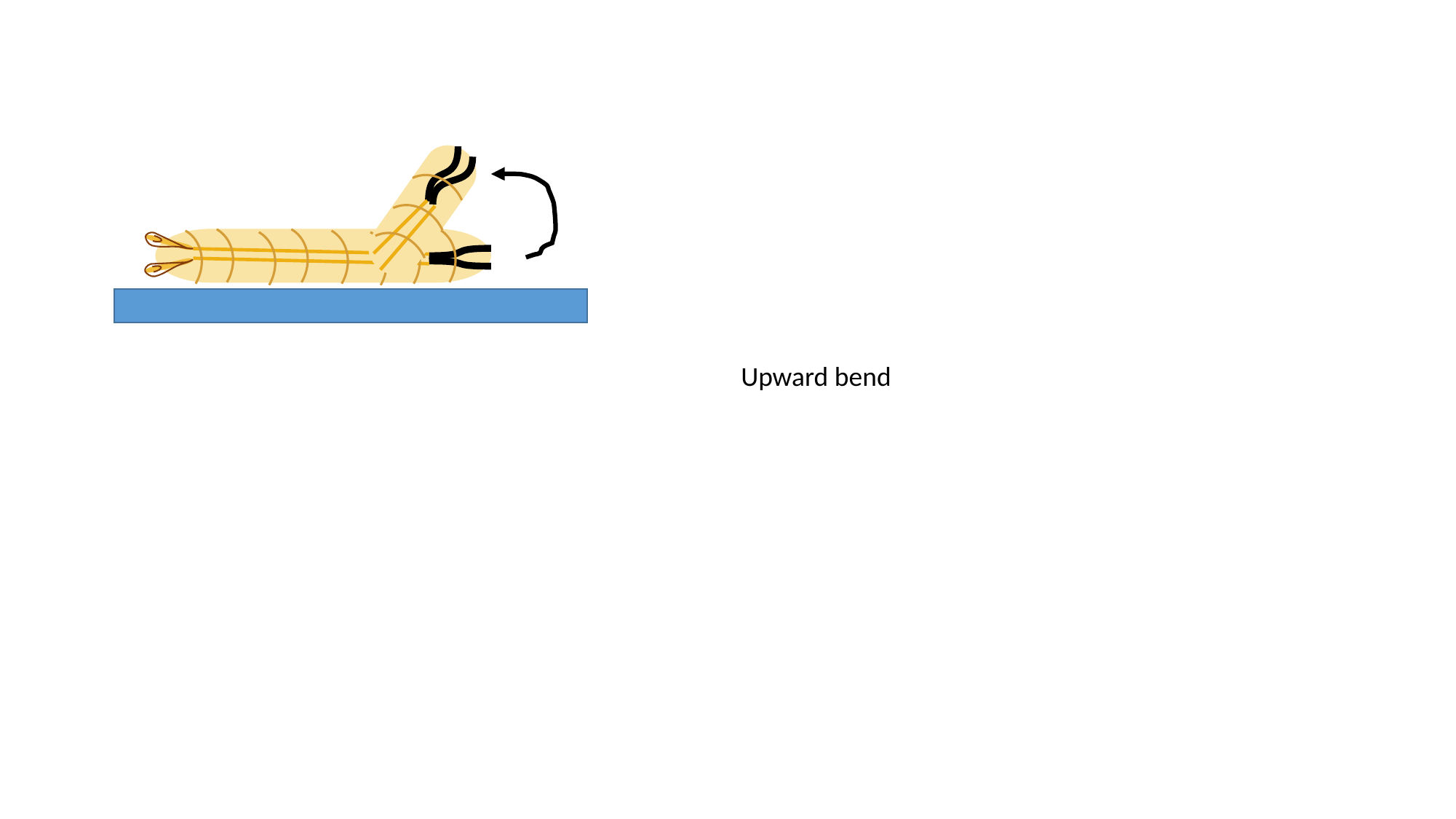

Upward bend

## Slide 6
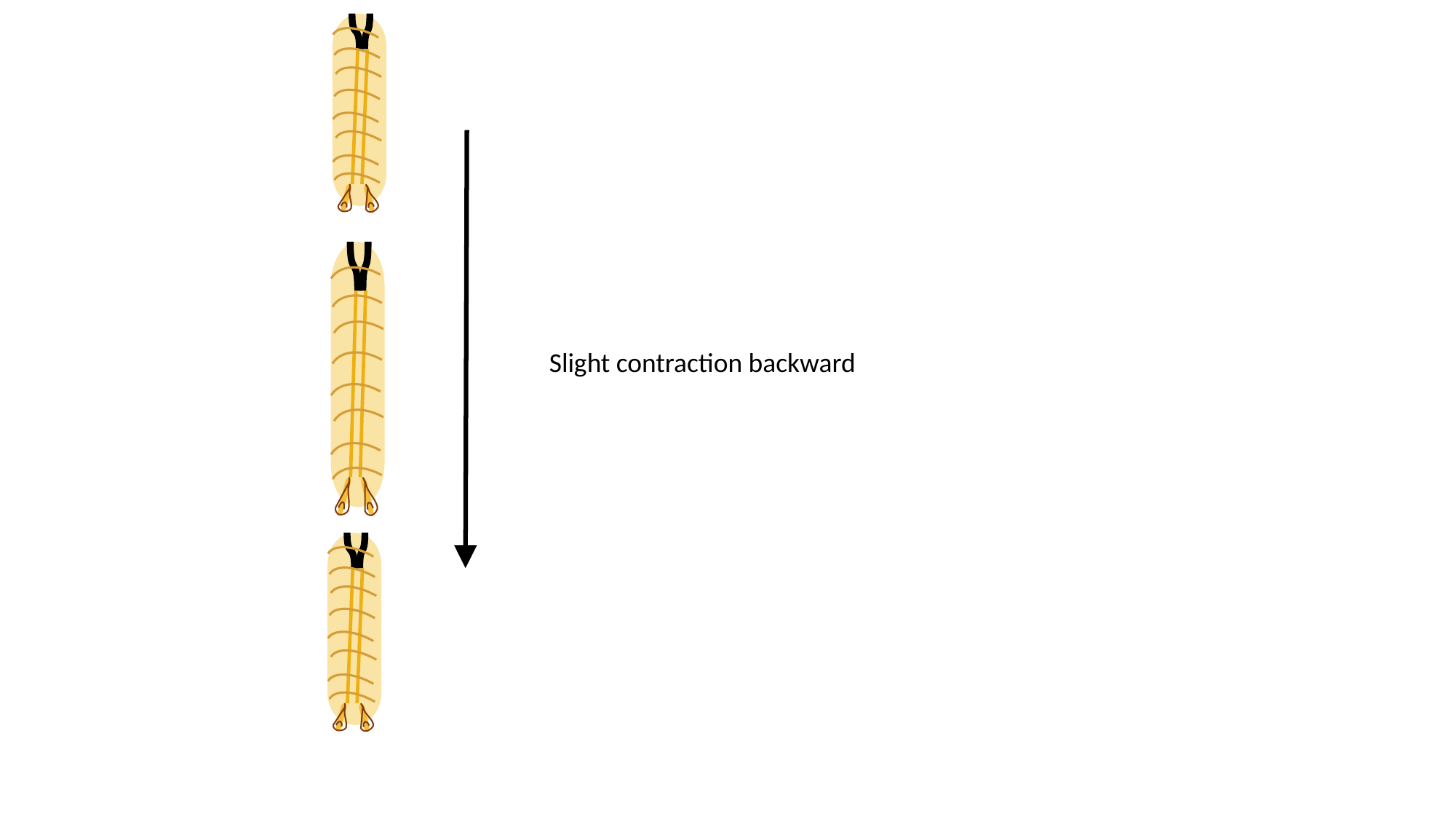

Slight contraction backward

## Slide 7
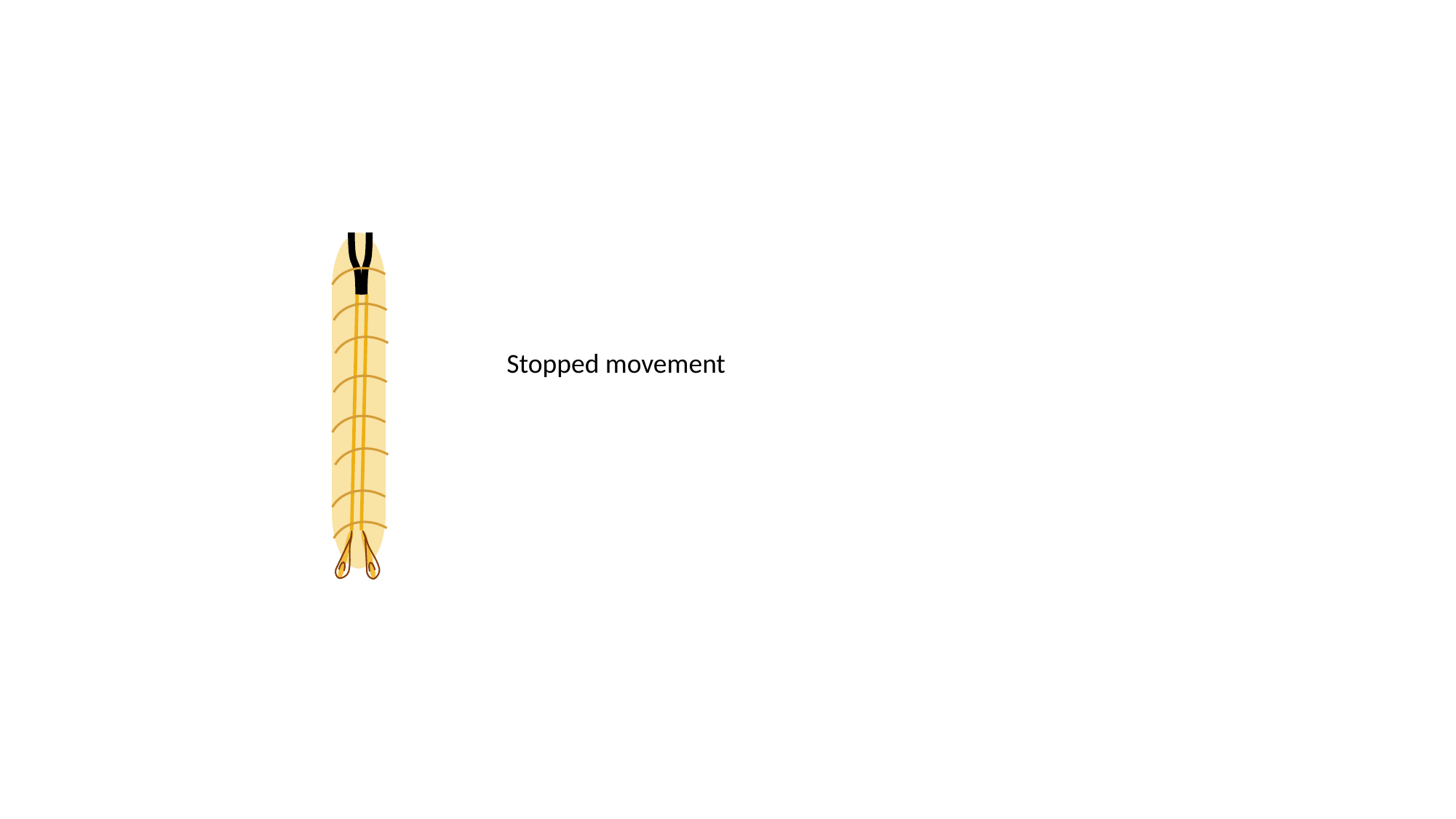

Stopped movement
